# Adding a new dimension to investigations of early radiolarian evolution

**DOI:** 10.1038/s41598-019-42771-0

**Published:** 2019-04-23

**Authors:** Sarah Kachovich, Jiani Sheng, Jonathan C. Aitchison

**Affiliations:** School of Earth and Environmental Sciences, The University of Queensland, Brisbane, QLD 4072 Australia

**Keywords:** Evolutionary ecology, Ocean sciences

## Abstract

Knowledge of the detailed architecture of the earliest radiolarian microfossils is key to resolving the evolution and systematics of this important group of marine protozoans. Non-destructive methods for observing the complexity within the internal structures of their siliceous skeletons have long eluded paleontologists. By developing methodologies that overcome some limitations of existing micro-computed tomography (micro-CT) we demonstrate a technique with potential to provide new insight into their evolution. Using 3D micro-CT data to generate models for six well-preserved siliceous radiolarian skeletons from the Middle Cambrian Inca Formation in far north Queensland, Australia and the Middle Ordovician Piccadilly Formation, in western Newfoundland, Canada, we can reconstruct phylogenetic relationships amongst some of the earliest radiolarians. Better knowledge of early radiolarian morphologies clarifies the vital function of internal structures and hierarchical diagnosis across a range of taxonomic affiliations.

## Introduction

Micro-CT has been adopted in fields as diverse as the mineralogical, biological, biophysical and anatomical sciences. Implementation in paleontology has been steady but has not displaced more traditional imaging methods in spite of its often-superior performance. We document methodologies that overcome some of the limitations to the micro-CT method and thereby progress, previously stagnant, research in radiolarian evolution and systematics.

With new insight on early radiolarian morphologies, we review the vital function of the internal structures and its hierarchical diagnosis of a range of taxonomic affiliations. Moreover, we demonstrate the challenges when working with microfossils close to the resolution limitation of the machine and provide procedures to reduce artefacts and analyse micro-CT data sets. By identifying useful methodologies for application of this powerful technique we seek to clarify choices for users wishing to apply micro-CT to other biological and paleontological research.

Polycystine radiolarians have the ability to fix intricate, siliceous (SiO_2_ × nH_2_O) skeletons, which can be subsequently moulded by the radiolarian into a wide variety of complex architectures without the loss of strength. Centrifugal growth of the skeleton is essential allowing the living cell to increase in size over its lifespan^1^. The relevance of the number of shells, skeletal spines and their connections can be investigated from a phylogenetic point of view to differentiate homoplasies from homologies. A large number of radiolarian groups are impossible to recognise simply on the basis of their external features because superficial spherical similarities among many taxa are a product of convergent morphologies, complimentary to their planktonic adaptation. As a result, many anomalies exist in differentiating between different spherical forms using traditional classification systems. Amongst early classification systems^2^, failure to distinguish true higher level homologous features has resulted in artificial lengthening of estimated radiolarian age ranges misleading many to consider that radiolarians to have little biostratigraphic potential^3^. Nonetheless, radiolarians have since proved their stratigraphic utility which has had significant implications in the geological sciences especially plate tectonics where they are widely used to constrain the timing of development of ancient ocean basins. Difficulties in refining the biostratigraphy of older radiolarians are commonly related to the typical states of preservation, where radiolarians partly or totally lose their transparency making traditional observation using simple transmitted light optics difficult^4–6^.

Spherical radiolarians represent a hitherto under-utilised but potentially valuable biostratigraphic tool. Much of this depends upon better understanding evolutionary changes to complex internal details of their mineralised skeletons. The main issue is that even though radiolarians are common in the geological record, our current knowledge cannot be simply arranged from “radiolarian ancestors” to “modern species” due to considerable preservation bias between sites and the absence of consistent taxonomy and nomenclature. In many samples, the majority of the spherical taxa recovered from Mesozoic and Paleozoic samples are simply too poorly known, taxonomically, to be employed in biostratigraphy.

Because homeomorphy is common among radiolarians and the values of most morphologic traits are still uncertain, a great deal of basic research is needed to better understand the taxonomic importance of these characteristics in order to find an appropriate place for each species in a natural taxonomic system. The basic premise for a recent family level classification scheme in the major review by Dumitrică in De Wever *et al*.^7^, covering all Phanerozoic radiolarians, is the notion that their internal morphology has a vital function throughout the life of an individual. This classification system is in part based on claimed homologous details of structure in the innermost part of many radiolarian skeletons and therefore there is a constant relationship with the morphology of the nucleo-axopodial complex^8^. It is assumed that the innermost portion of the skeleton is the first part secreted during ontogeny and is therefore under biogenetic law, the most conservative feature. This hypothesis is supported by many authors^9–13^. Moreover, it is only at the species level, that observation of the surface topography can be used for discrimination of individual taxa.

The phylogenetic relevance of the internal morphology of a radiolarian skeleton (generally less than 50 µm) for the foundations of evolutionary studies has previously been recognised but has not yet been fully used in radiolarian taxonomy due to limitations of available technologies for their observation. Classical approaches have taken us so far and continue to be useful. However, the pursuit of new biological insights into spherical radiolarians requires a systematic approach with high spatial resolution and a large depth of field, which can be achieved with the micro-CT method.

## Results

**Figure 1:**
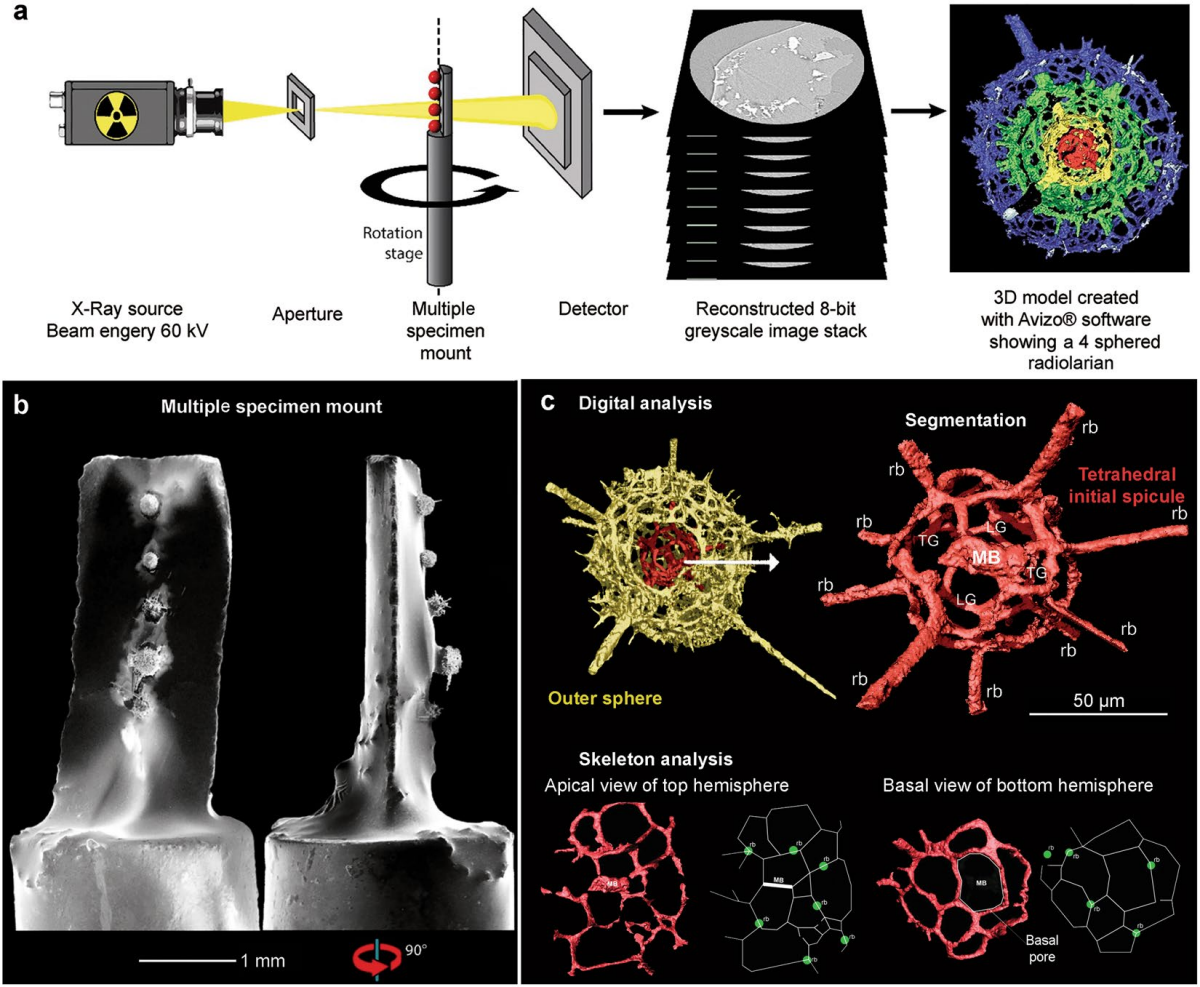
Workflow for detailed radiolarian taxonomic study with the micro-CT. **(a)** General overview of the micro-CT scanning and reconstruction. **(b)** Scanning electron micrographs of the multiple specimen mount, where alignment of specimens is along the central rotational axis. **(c)** micro-CT models of processed data to show the digital analysis of the internal feature. Planar extraction of the apical view and basal view of the inner sphere. MB = median bar, rb = radial beam, TG = transversal gate.

**Figure 2:**
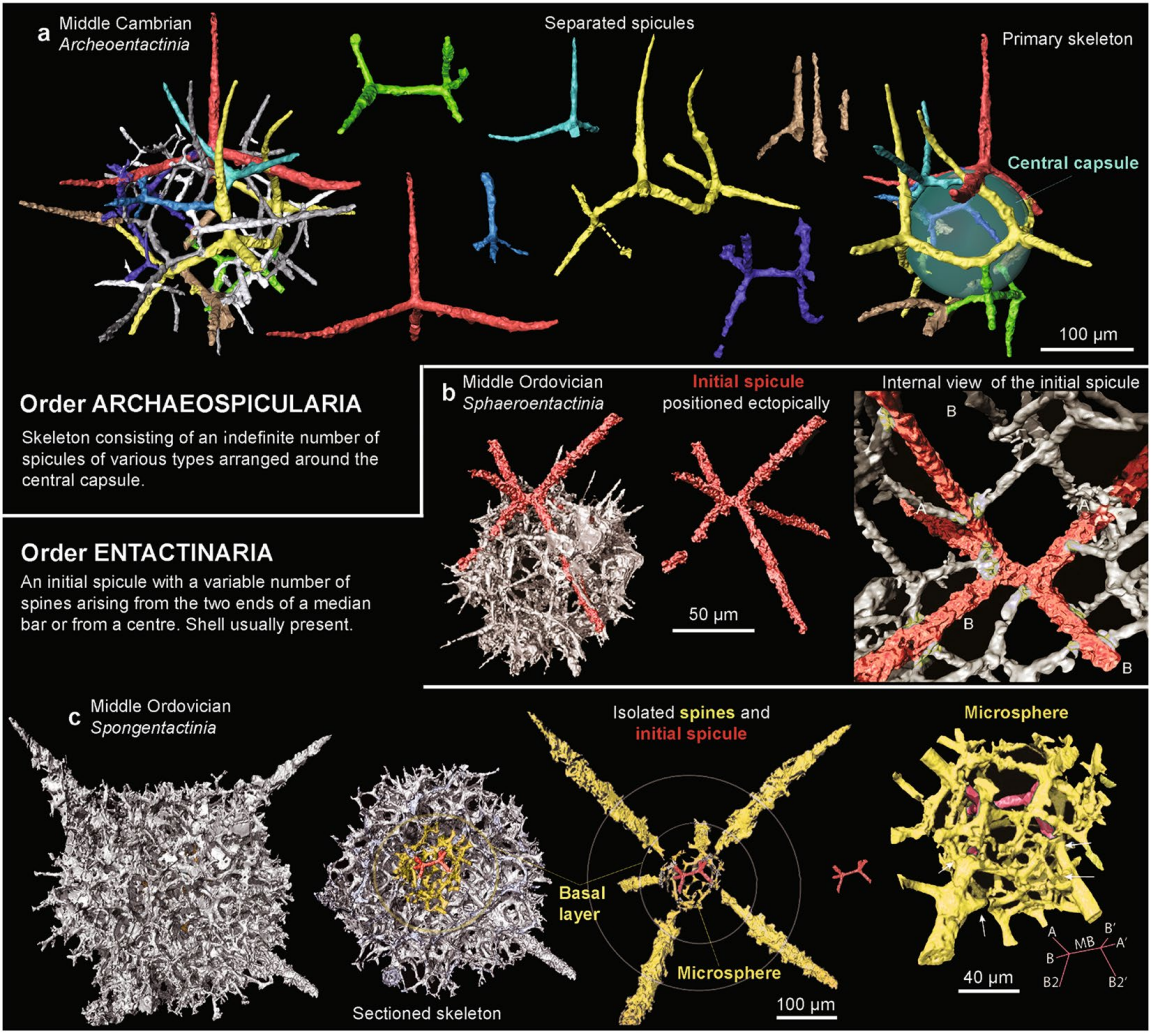
Micro-CT models of various radiolarian groups illustrating the initial spicule (red) in relation to their heteropolar skeleton. Each segment is shown at the same scale unless indicated. **(a)** Segmented spicules of *Archeoentactinia incaensis* Won from the Middle Cambrian Inca Formation, far northwest Australia. Green sphere digitally inserted to test the sphericity of the central capsule. **(b)** Ectopically positioned spicule of *Sphaeroentactinia* sp. aff. *S. integrata* (Maletz and Bruton) from the Middle Ordovician Piccadilly Formation, western Newfoundland. Fusing points of the meshwork and initial spicule illustrated. **(c)** Internal features of *Spongentactinia armillata* (Nazarov) from the Piccadilly Formation. Six-rayed internal spicule with a median bar (red) is digitally segmented from the meshwork (grey).

**Figure 3:**
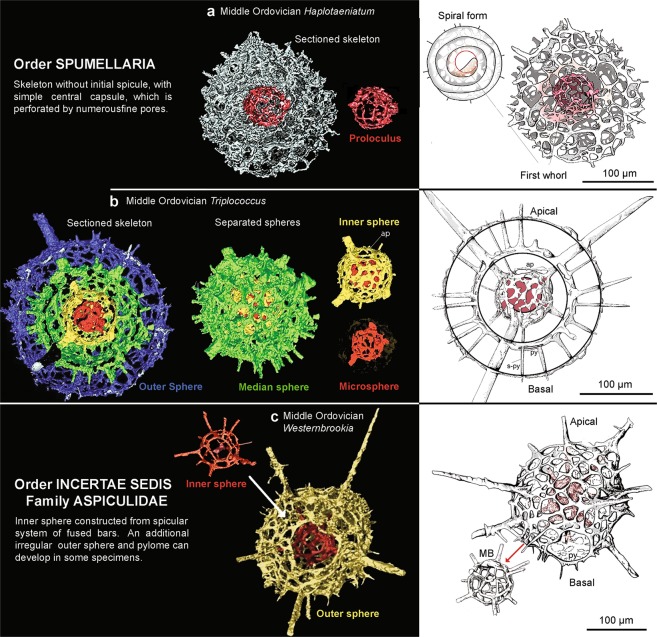
A compilation of line drawings and micro-CT models of various radiolarians from the Middle Ordovician Piccadilly Formation, western Newfoundland with the innermost sphere highlighted (red). Each segment is shown at the same scale. **(a)** micro-CT models of *Haplotaeniatum* sp. cf. *H. vertigospongum* (Jones and Noble) capturing the complex spiral growth of the skeleton. **(b)** *Triplococcus acanthicus* Danelian and Popov demonstrating a complete morphological observation of the relationship between spheres and spines without compromising the integrity of the specimen. **(c)** Undescribed new species of the genus *Westernbrookia* demonstrating a primitive tetrahedral initial spicule similar to that of Triassic^27^.

A systematic investigation was undertaken of thirty-one micro-CT scanned specimens (six presented here) extracted from fine-grained limestones collected at two known lagerstätten deposits in far northwest Queensland, Australia and western Newfoundland, Canada. A single specimen from a Middle Cambrian (Epoch 3) calcareous concretion within black micrites of the Inca Formation, Georgina Basin (19°44′15.7″S, 138°53′51.9″E) was examined. Five additional radiolarians recovered from dark grey nodules within fossiliferous peloidal limestones of the Middle Ordovician (middle Darriwilian) Table Cove Formation, at the Piccadilly Quarry, Port au Port Peninsula (48°35′30.0″N 58°55′13.3″W) were also investigated. To obtain cleaner datasets with micro-CT, where X-rays are diffracted more precisely, radiolarians were chemically extracted from their host rock. This facilitated a crucial reduction of X-ray artefacts allowing clean and easy segmentation. The results provide new insights allowing evaluation of morphological characters in three dimensions (3D). The homologous traits reflected in the internal physiology of these radiolarians, are false coloured red in all micro-CT models (Figs 1–3). In micro-CT studies of radiolarians an obvious experiment is to explore the possibilities of the accurate description of the 3D structure of radiolarian tests, with particular emphasis on pore structure and connectivity^14–16^. By observing the 3D structure of often delicate and uncommonly preserved internal features improved knowledge of the taxonomy of spherical radiolarians can be achieved and this will facilitate better understanding of their evolution and, as a consequence, their biostratigraphic utility can be realised.

### Considerations for micro-CT scanning at the highest resolution

Since the first micro-CT publication^17^ of a 3D scan of a small tropical snail, with a voxel size of about 50 µm, the lower limit of resolution has greatly improved. However, because radiolarians average between 63 and 240 μm diameter, and the objective of this study was to observe smaller internal detail, the machine (Xradia Versa 500) was operated at its maximum resolution limit. We emphasise here that high resolution scans are more sensitive to problems that are not normally an issue for general micro-CT scanning.

An overview of our approach (Fig. 1) highlights our strategy to minimise failed scans and scanning artefacts in the analysis of the fine details at a high resolution. During data acquisition, beam energy was lowered to 60 kV to compensate for an increase in phase contrast at the cost of developing scanning artefacts^18^. Lower beam energy was crucial for obtaining sufficient contrast at this resolution in order to observe the internal features of radiolarian specimens. As a result of lower beam energy, beam-hardening and edge-enhancement artefacts may develop because the X-ray beam attenuates as it passes through an object^18^. All of the radiolarian skeletons scanned generated images with very bright edges due to this X-ray artefact, which can make a body of uniform density appear as though it is not always homogeneous and has a low-density core. These artefacts are unavoidable but can be reduced in the post scanning digital clean.

Thermal flux is not normally an issue for micro-CT scanning but as this study approaches the lower limits of the machine, small fluxes pose threats to the experiments. It is crucial that the specimen does not move during scanning, especially when working below the 1.0 µm pixel resolution. Even a minor shift can ruin the data, or necessitate extremely time-consuming corrections. Mounts designed to fix multiple specimens were developed and these ideally suited scanning conditions without sacrificing the integrity of the specimen. It was found that a single mount was more susceptible to unsuccessful scans, as slight thermal flux in the chamber during specimen exchange can be too great and cause a scan to fail. Moreover, a multiple specimen mount not only decreases the number of failed scans, due to thermal flux, but also saved time during specimen exchange as multiple scans can be preloaded and the machine left to run. In this technique, radiolarians were vertically mounted along the central rotation axis of the mount, which aided in automated programming.

The mount material and mounting media were also carefully selected. Preferred mounts and mounting media have relatively low density compared to that of the siliceous radiolarian specimen (2.65 g/cm^3^). This helps to reduce beam-hardening artefacts and increase contrast sensitivity. An ultraviolet (UV) curing resin, Norland optical adhesive 61 (NOA61) was chosen as the favoured mounting medium for four reasons: (1) it has low density compared with silica (NOA61 = 1.231 g/cm^3^); (2) it remains a fluid until cured with UV light; (3) it does not become brittle when it hardens; (4) it is readily available as it is the common mounting medium radiolarist use to make slides for transmitted light observations. Although removal of the specimen from the mounting medium was not attempted in this study, cured NOA61 substrate can be removed by soaking in a chlorinated solvent such as dichloromethane, catalysed by methanol and concentrated ammonia^19^.

Acrylonitrile butadiene styrene (ABS) plastic recycled from the offcuts of a fused deposition modelling 3D printer was selected for a multiple specimen mount because of compatibility of dimensions with the micro-CT mount holders, low density (1.07 g/cm3), the ease of carving and accessibility. ABS plastic was carefully carved under a reflected light microscope with a surgical scalpel to make a platform for the specimens, which were mounted near the central axis of rotation. While the mount was horizontal, NOA61 was applied to the surface of the active mount and specimens were transferred from a micropaleotology specimen slide mount. Once cured with UV light, the ABS mount can be stored in an upright position.

### Illustrating the third dimension

Representative diagrams that illustrate different possible interpretations of skeletal architecture are commonly presented in studies that describe radiolarians based on observations made using scanning electron microscopy (SEM). The inability to fully observe specimens using this technique has resulted in misidentification of numerous taxa, or incorrect provisional assignment to particular genera or families^20,21^ thereby impacting the usefulness of some groups of radiolarians in biostratigraphic investigations and evolutionary studies. Good illustrations are critical for the study of Lower Paleozoic radiolarians, as many forms are not currently well documented and still require systematic description. 3D digital models offer continuous availability of type material to all potential users both instantly and simultaneously. However, micro-CT datasets take months to analyse and without proper descriptions and clear illustrations it is difficult to know the true extent of variability within and between groups.

High-resolution micro-CT data sets were examined in this study in order to assess both the morphology of complete specimens as well as to focus on important segments of some taxa. Avizo® software was chosen because of the appropriate suite of commands, flexibility of its graphical user interface and end user-friendly display. The type of imaging enhancement and segmentation methods used also has a significant impact on the results. Colour schemes are often overlooked but are an important tool that can be used for the visualisation of scientific graphics and communication. Although colour schemes are highly subjective, they can be used to assist the reader to easily understand the data. Segmented spheres and spicules need to be carefully assigned to an appropriate colour scheme. Our colour scheme is used to highlight important aspects of the data and splits them into hierarchy of categories. For example, choosing colours that link taxonomically important features, such as a red microsphere and initial spicule, helps the author highlight the most crucial features to a specimen (see Figs 2 and 3 for examples of illustrations achieved from micro-CT data that can be applied to taxonomy studies). Furthermore, by creating a split-complementary scheme the colour choices are also accessible to those with colour vision deficiency.

### Potential for addressing problems with the current classification system

Micro-CT imaging can help us to identify natural relationships between complex higher-level morphologies of Paleozoic radiolarians and improve our understanding of early life evolution. Presently, the orders Entactinaria (also Archaeospicularia, Albaillellaria, Latentifistularia and Nassellaria) and Spumellaria are differentiated based on the presence or absence of an initial spicule, respectively^18,22,23^. Currently, the relationship of the initial spicule to the first sphere in entactinarians is important for taxonomy at the family level and can be positioned centrally (e.g. Entactiniidae and Pylentonemidae) or eccentric (e.g. Secuicollactidae, Archeoentactiniidae, Pentactinocarpidae and Rhizosphaeridae). The six-rayed, ectopically positioned spicule is a stable feature (Fig. 2). The arrangement of the six rays and the position and length of the median bar (MB) is rarely variable at the species and occasionally the genus level. Won^24^ found similar limits to variation in internal skeletal structure of Devonian entactiniids at both the species and genus levels.

Polarisation of the skeleton observed in the majority of the micro-CT scans suggests that our understanding of these early groups needs revision. This is not simply a single or homologous character in radiolarian evolution, but a general possibility and trend in many groups of radiolarians. It develops independently and is expressed in various ways and in different skeletal features. However, the type of polarisation appears to be unique, occurring as an eccentrically placed spicule or sphere, apical and basal pores, polarised spines, internal spicule or as a pylome.

By digitally inserting a sphere into the central cavity of the 3D models, sphericity of the test can be analysed (Fig. 2a). In the Middle Cambrian *Archeoentactinia incaensis* Won the main skeletal framwork is perfectly spherical, where the smaller meshwork is above this sphere. This confirms a stepwise growth of the skeleton as ontogeny proceeds. The homologous trait of the primary skeleton can now be compared to other genera and families in this group of spherical radiolarians.

Amongst spumellarians the initial element is the innermost sphere (generally < 50 µm) and is restricted to the centrally placed sphere from which the outer spines originate. In some families in the order Spumellaria (e.g. Haplotaeniatidae, Actinommidae, Pyloniidae, Litheliidae) initial growth begins with a microsphere (termed proloculus) where radial bars connect the successive, concentric spheres or consecutive whorls to form a spiral (Fig. 3a). Micro-CT scans of the microsphere of representatives from the inaniguttids (Fig. 3b) show that the proloculus is very distinct from that in the haplotaeniatids. The proloculus is latticed, where its sphere is constructed of connecting bars. The site where the first whorl develops is densely latticed. In the inaniguttids, a well-developed shell and pores were observed. The fusing of spinules, to create a defined spherical-wall, is a heterogenetic feature that has occurred many times, even within the entactinarians (e.g. Proventocitiidae, Protoentactinidae and Echidninidae).

In our investigation of the Middle Ordovician aspiculids, spicular elements similar to those of early Mesozoic taxa (e.g. tetrapoidal spicular system)^25^ were observed in some specimens. Currently, comparison is difficult as it is uncertain whether the microsphere of Triassic forms is homologous to the microsphere observed in early Paleozoic species (Fig. 3c). Such features are currently unknown through the late Paleozoic and the apparent long absence of this kind of spicule structure in the fossil record is curious. It is regarded herein as mostly likely a result of convergent evolution. The variability of the inner spicular system within the aspiculids remains difficult to understand and place with the context of other Paleozoic radiolarians so this group is left as *incertae sedis*.

### Application of reconstructions to understanding evolutionary lineages

Previously, information regarding the initial element in many taxa has been insufficient to allow testing of proposed major linages. These radiolarians observed in this study provide important data, filling the gap in our understanding between Paleozoic and Cenozoic initial spicule-bearing spherical radiolarians. Furthermore, realising that Spumellaria are specialised members of the Entactinaria raises the interesting question: what happened when the first spumellarian evolved from its entactinarian ancestor, and why are Spumellaria are more highly successful. — there are more extant species in the spumellarian branch than in the rest of enatactinarians, what made them more successful after the end of the Triassic.

**Figure 4:**
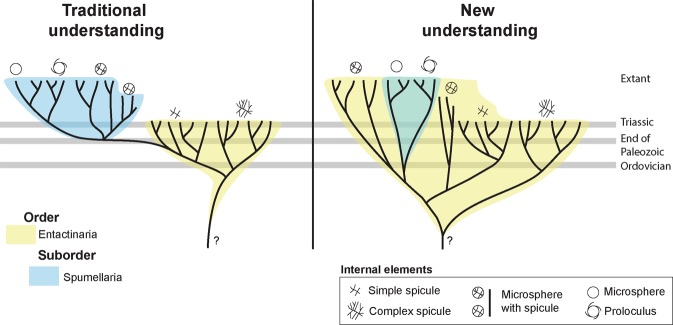
Entactinarian phylogeny as inferred from internal morphological studies. In our new understanding, spumellarians are a Suborder within the Order Entactinaria. The nature of internal elements needs to be treated as a family and genus level feature.

The anomalous situation where some Spumellaria appear more closely related to some species of Entactinaria than other spumellarians in the Mesozoic has long challenged paleonotologists^7,26–31^. Some spumellarians observed in this study have been observed with an initial spicule, but the phylogenetic relationship of this feature is uncertain as no intermediate forms exist that show connections to the Entactinaria. The presence of homologous elements of the internal framework and its rudiments in the majority of radiolarians suggests the monophyletic origin of the spumellarians within the entactinarian group (Fig. 4). This idea has already been favoured by a some researchers^24,32–35^. Furthermore, our observations constrain the maximum age of the Spumellaria to the Middle Ordovician.

## Discussion

The specificity, sensitivity and accuracy of this imaging modality in detecting the internal features allows trait characteristics shared between various high-level taxonomic groupings of radiolarians to be accurately assessed. This will eventually lead to a natural taxonomy by eliminating many anomalies amongst existing classification schemes, which currently exist to differentiate amongst spherical forms of the Paleozoic radiolarians.

Our strategy differs substantially from conventional micropaleontological approaches. Omnidirectional observation of the internal and external structures of single specimens provide new data with which to test taxonomic hypotheses. Using micro-CT observation, the phylogenetic relevance of various internal features of complex radiolarians can now be explored in more detail. We have presented examples to illustrate the value of this method both in challenging the *status quo* and to generate new hypotheses.

Application of micro-CT for detailed, virtual taxonomy will accelerate discovery in a range of new research opportunities from taxonomy, phylogeny, biostratigraphy to geology. The siliceous skeletons of radiolarians offer major advantages in the identification and biostratigraphic studies of early eukaryotes, particularly where calcareous microfossils might have suffered dissolution. Furthermore, radiolarian biostratigraphy is particularly important for constraining the ages of remnants of sediment covering ocean crust hat is incorporated into subduction complexes that are otherwise unfossiliferous and difficult to date. Thus, any improvements to biostratigraphic resolution have the potential to improve our understanding.

## On-line Methods

Radiolarian skeletons potentially can exist in most fine-grained marine deposits including soft clay mudstones, calcareous oozes or marls, hard shales, limestones and cherts. A variety of forms can be recognised in well-preserved, lagerstätten material, which provided us with the opportunity to investigate an entirely new assemblage with the aid of micro-CT. The following information outlines basic procedures and techniques used in this investigation of Paleozoic spherical radiolarians including initial SEM analysis, micro-CT mounting, scanning and digital processing.

### Assessment of material for micro-CT analysis

**Figure 5:**
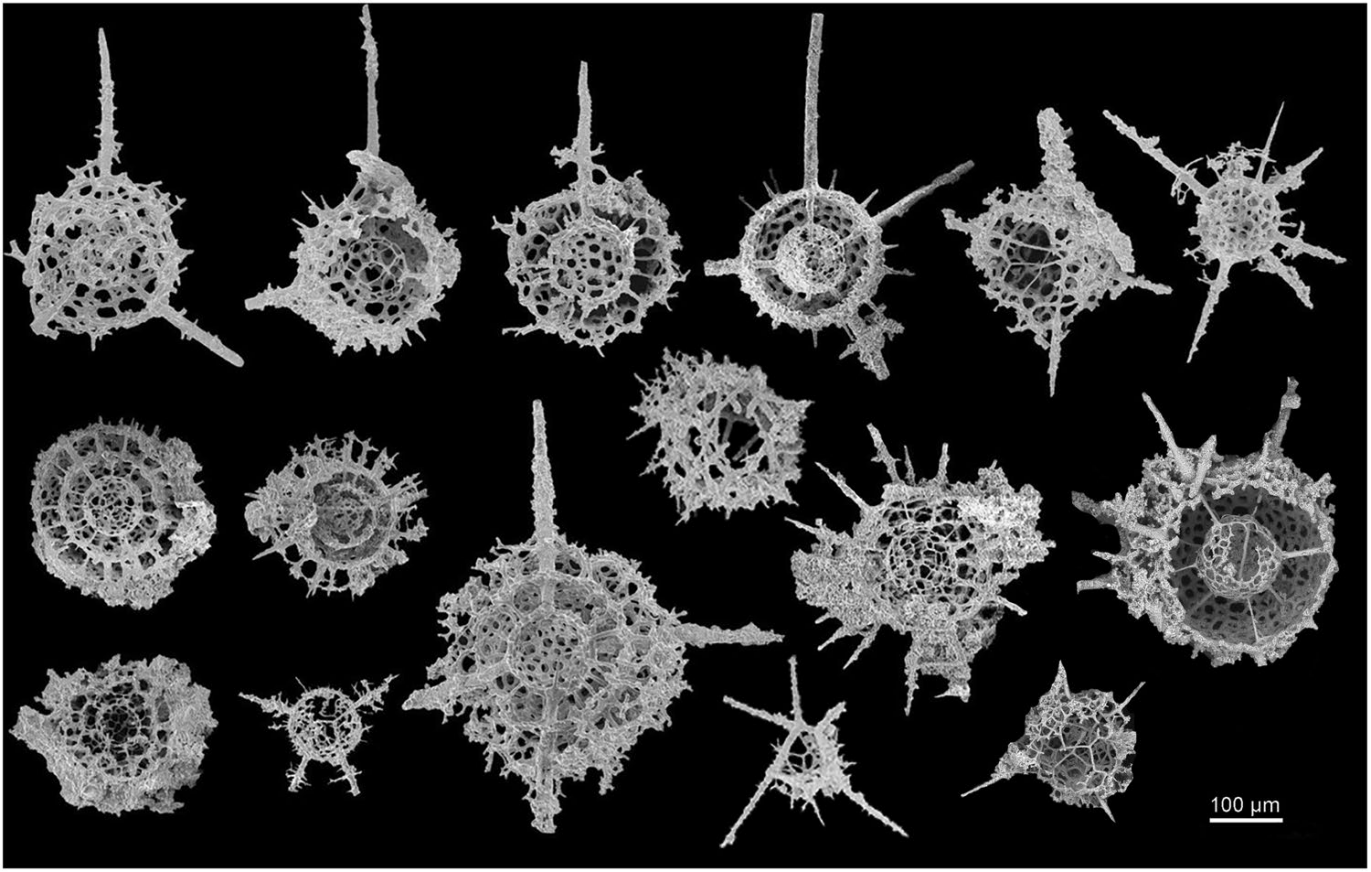
Initial examination of the Middle Ordovician samples from the Piccadilly Quarry, western Newfoundland, Canada was undertaken with the SEM to assess the preservation of the internal features in the broken specimens. Scale bar: 100 μm.

An initial assessment with the SEM is used to test if the micro-CT method is appropriate (Fig. 5). Carbonate samples were broken down to approximately 5 cm^3^ pieces and treated with 10% acetic acid, for 50 days — topped up if effervescence subsided. Samples required slightly varying concentrations of acid in order to achieve a gentle effervescence because an overly vigorous reaction would cause tests to be easily broken. Moreover, samples needed to be completely submerged at all times to stop the precipitation of calcium acetate crystals that can damage radiolarian tests.

After acid treatment, the residues were wet sieved through a 250 μm sieve and microfossils were collected on a 63 μm sieve. If aggregation occurred, such as the clumping of grains and adhesion of clay flakes to individual fossils, the residue received a treatment with a deflocculant (Calgon 5% concentration). Residues were individually picked using a fine-tipped paintbrush under a reflecting light microscope.

It is essential to understand early Paleozoic radiolarians faunas in the context of other fossil groups in order to establish a precise and useful radiolarian biostratigraphy that permits an integration between standard biozones. The material from the Inca Formation, in the Georgina Basin comes from a well-studied locality where trilobite biostratigraphy has provided a reliable Middle Cambrian (Epoch 3) (Templetonian *Triplagnostus gibbus* and Undillan *Ptychagnostus punctuosus* zones) stratigraphic control^36,37^. The radiolarian-bearing samples from the Table Cove Formation at the Piccadilly Quarry also yielded a co-occurring age-diagnostic conodont assemblage. A middle Darriwilian age (late Dw2) age was determined by the recovery of biostratigraphically important pectiniform taxa such as *Histiodella kristinae* and the genus *Polonodus*^38^.

### Micro-CT preparation

To determine the preservation of the delicate internal structure before selection for micro-CT, specimens were in water and observed at low magnification in reflected light using a stereo microscope. By immersing the test in a droplet of water, the silica becomes slightly transparent and pyrite and any quartz infilling becomes more apparent.

Acrylonitrile butadiene styrene (ABS) plastic recycled from the offcuts from a 3D printer was selected for a multiple specimen mount because of its low density (1.07 g/cm3) and the ease of carving. ABS plastic was cut into 2 cm long mounts, which were then carefully carved with a surgical scalpel to make a platform for the specimens. While the mount was horizontal, Norland optical adhesive 61 (NOA61) was applied to the surface of the active mount and specimens were transferred from a micropaleontology slide to along the central axis of rotation. Once cured with UV light, the mount was stored in an upright position. Each mount was imaged for spatial reference then placed into a clamp holder for scanning. It is important to know where the specimens are on the mount for ease of navigation when setting up the micro-CT scans.

### Micro-CT data acquisition

The Xradia Versa 500 micro-CT scanner at the University of Queensland Julius Kruttschnitt Mineral Research Centre has a wide range of magnifications, down to <0.7 µm. Very simply, micro-CT methodology produces a series of radiographs of a specimen taken at various orientations which are then stitched together to digitally reconstruct the specimen.

The fine detail of internal features and long external spines were often omitted from the scan in order to increase the resolution. Preferred scans parameters were with a beam energy of 60 kV and X-ray source current of 166 μA. This allowed us to obtain optimum exposure and maximum contrast^39^. Preferred scans ran for four hours. Volume reconstruction software integrated with the Xradia 500 instrument then were saved in TIFF format for processing using Avizo® software. The images provided a resolution of 0.42 μm and the data were visualised and analysed using Avizo® 9.3 software.

### Artefact reduction and analysis

Beam-hardening and edge-enhancement artefacts commonly caused obstructions in the specimen and produced poor results with all the available types of density thresholding. Using automatic thresholding methods, the accuracy of statistical algorithms for quantitative analysis of the 16 bits of grey scale information, per voxel, can render the data problematic. However, the computer-assisted manual segmentation technique is complicated and time-consuming. We adopted the philosophy that the rate of segmentation is less important than the greater accuracy that can be achieved through a supervised manual segmentation process. Although automated thresholding is the less suited than the manual approach, it was still needed initially to remove the lower density constituents (air, ABS mount and NOA61 glue) of the dataset.

Noise reduction is not trivial and different algorithms exist for achieving clean micro-CT data. Critical judgment and careful monitoring is required during the development of models as data is subject to segmentation error because of complex morphology. The method of using a simple “Median” filter pre-segmentation and the “Fill” module post-segmentation was commonly adopted here^40^. In most cases, a simple median filter was employed to reduce the white noise in the data. However, an advanced edge-preserving smoothing algorithm had to be applied when the median filter blurred the data too much and caused indistinguishable contrast between areas of interest^41^. The “Fill” module was applied to ensure the low-density artefacts were removed.

Segmentation allows the model to be virtually dissected and viewed at any desired angle, offering a unique opportunity to identify the nature of every component. This is particularly important with models of radiolarians as some of their features are repetitive and the areas of interest are not visually accessible or clear without data manipulation. Information regarding pore shape and size was only available after segmentation. This was achieved by defining the proportion of the image corresponding to the feature of interest, such as spines, microspheres or spicules. Manual segmentation required persistent input by the operator with image filters, intensity thresholding, morphologic filters, and manual outlining features.

In Avizo®, the specimens were segmented twice, first to clean the model by separating it from any air, then from the glue and undigested matrix using the ‘Label-field’ segmentation editor. This automatically converted data into discretised (binary) images. A section of the specimen was selected with the ‘Magic wand’ tool and the threshold was adjusted to include only the density related to silica. This was then added to a new label field in which mechanical segmentation was applied.

The model was then segmented in order to study internal features. To empirically establish where internal spicules and connections were present, the outer topology was carefully stripped away. The favoured algorithm to isolate different spheres utilised full 3D processing, which enabled the operator to overlay and subtract geometrical sphere, of varying sizes, within the model. Started on the internal sphere and progressively working towards the outer sphere. This produced an inverse data set. At each step, the data were duplicated in order to connect an “Arithmetic” module between data to obtain the isolated skeletal sphere. When adding input data from two or more sources the combined model sometimes generates artefacts, when two materials touch and share part of the surface. When adding the “Arithmetic” expression should always be set to subtract any voxels with a value of 2 instead one 1. To analysis the pore connectivity and determine basal spine generation on the initial sphere, the microsphere was segmented in half and the two hemispheres were flattened to create a planar extraction graph (Fig. 1c). This procedure follows that of Matsuoka *et al*.^12^ but the method here manipulates the digital data. From planar graphs, pore frames and networks can be accurately determined together with relationships to the radial beams.

The last step was to render 3D volume displays using the “Generate Surface” module to generate and edit surfaces. The smoothing level was set to 1 to avoid distortion of the models. The “Surface Generate” volume rendering module integrated the cross-sectional area of each vessel along its length. Transparency adjustment of segmented features made it easy to toggle between solid and transparent layers during observations. To evaluate any errors associated with segmentation, SEM and TLM images, as well as the raw CT slices, were assessed to provide a uniform basis for consistent comparison. Digital separation using Avizo® software allowed complete morphological observation of the internal structure of the specimen without compromising the integrity of the specimen.
